# Challenges and Stakeholders' Views on Achievements of Multi-Sectoral Governance in Improving Child Nutrition in Buhigwe District, Tanzania

**DOI:** 10.24248/eahrj.v7i2.733

**Published:** 2023-11-30

**Authors:** Mziraya Zidikheri, Frumence Gasto

**Affiliations:** a School of Public Health and Social Sciences, Muhimbili University of Health and Allied Sciences, Dar es Salaam, Tanzania; b Department of Development Studies, School of Public Health and Social Sciences, Muhimbili University of Health and Allied Sciences, Dar es Salaam, Tanzania

## Abstract

**Background:**

In recent years, Tanzania has made good progress in addressing the problem of chronic malnutrition in children, but the levels are still unsatisfactory, at almost three in ten of its children are stunted. The government of Tanzania has taken significant measures to establish and strengthen multi-sectoral governance as part of national efforts to reduce the unacceptably high level of malnutrition. However, few studies have qualitatively documented stakeholder's perspectives at sub-national and community level with regards to performance of multi-sectoral governance in improving nutrition status of children in Tanzania.

**Objective:**

This study aimed to explore the achievements, facilitating factors and challenges of multi-sectoral governance in improving child nutrition in Buhigwe District Council.

**Methods:**

This was cross-sectional study, which employed qualitative method of data collection whereby semi-structured interviews were used to conduct in-depth interviews with members of the multisectoral steering committee for nutrition in Buhigwe Council and parents/caregivers of under-five children.

**Results:**

There is significant improvement in allocating funds for multisectoral governance interventions targeting under five children across sectors, improved inter-sectoral coordination and increased knowledge on feeding practices among parents/caregivers. Strong leadership and political commitment, inter-sectoral linkage and Presence of Non-Governmental Organizations (NGOs) supporting nutrition were identified as factors for improving child nutrition in the context of multisectoral governance. The issue of insufficient funding, inadequate spending of allocated funds, inadequate capacity, lack of cross-sectional financial mechanism and tools to collect nutrition information were raised by stakeholders as factors hindering the implementation of multi-sectoral governance in improving child nutrition.

**Conclusion:**

Smooth implementation of multi-sectoral interventions targeting under-five children requires strong multi-sectoral governance, which is supported by adequate spending of the allocated funds, strong leadership and political commitment, presence of NGOs supporting nutrition and inter-sectoral linkage among line sectors. However, key stakeholders including central and local government authorities should address the challenge of insufficient funds, inadequate capacity, lack of cross-sectoral co-financing mechanism and tools to collect nutrition information, which were reported as barriers to the implementation of multi-sectoral governance in improving child nutrition.

## BACKGROUND

For the past one decade, there has been a growing literature highlighting the importance of multisectoral governance for nutrition. The UN's Standing Committee on Nutrition defines “global nutrition governance” as the network of actors whose primary function is to improve nutrition outcomes through processes and mechanisms for convening, agenda setting, decision making (including norm-setting), implementation, and accountability.^1^ Child undernutrition, including stunting, is an important example of a global challenge that spans multiple sectors, including health, agriculture and food systems, water and sanitation, education and gender. Stunting is the impaired growth and development that children experience from poor nutrition, repeated infection, and inadequate psychosocial stimulation. Children are defined as stunted if their height-for-age is more than two standard deviations below the World Health Organization (WHO) Child Growth Standards median.^2^ Both strong evidences of the damage caused by undernutrition and the multiple benefits in reducing undernutrition led to the emergence of nutrition governance.^3^ The strong nutrition governance was associated with lower magnitude of stunting and underweight in low and middle-income countries.^4^

The Scaling Up Nutrition (SUN) Secretariat is a governance network established in 2010 that leads a global movement across 61 low- and middle-income countries to reduce malnutrition by 2030.^5^ The Government of Tanzania (GoT) signed up to the SUN movement in 2011 and further issued a ‘Presidential call to action for increased accountability in tackling the nutrition situation in the country. The Prime Minister's Office ordered the establishment of multisectoral steering committees for nutrition (MSCN) at each level of government, appointed regional and district nutrition officers and issued nutrition guidelines requiring districts to budget for the implementation of nutrition activities.^6^ In Tanzania, Council level committees are integral to nutrition governance, playing a key role in ensuring that nutrition policies and programs are implemented in alignment with the National Nutrition Strategy (NNS) of 2011/12-2015/16 which preceded the National Multisectoral Nutrition Action Plan (NMNAP) 2016 – 2021. The decentralization of nutrition interventions through council multi-sectoral nutrition steering committees on nutrition (CMSCN) provides Tanzania with a strong system for multi-sectoral nutrition governance.^7^ The CMSCN meets quarterly with members from key nutrition stakeholders including heads of nutrition sensitive sectors, nutrition focused Civil, Faith based, other non-governmental organizations (CBO, FBO & NGOs), the media, private sector, academic and research institutions depending on the agenda.^8,9^ Despite the government efforts in strengthening multi-sectoral governance at sub-national level, almost 3 in 10 of its children are still stunted, making the country home to the third highest population of stunted children in Sub Saharan Africa, after Ethiopia and Democratic Republic of Congo.^10^

Published studies exploring stakeholder's perceptions on multisectoral governance for nutrition at sub-national and community level remains low both in Tanzania and globally. In fact, many studies have provided evidence of the effectiveness of multisectoral programs in reducing chronic malnutrition.^6,7,12–16^ This study was an attempt to explore and analyze the achievements of multi-sectoral governance, facilitating factors and challenges hindering its implementation from the stakeholder's viewpoint to improve the nutrition status of children in Buhigwe District Council. The participants for this study were drawn from the Buhigwe District Council, which has a high number of malnourished children with low height for age which is high compared with that of Kigoma Region.^17^ The findings from this study also compliment the Joint Multisectoral Nutrition Reviews (JMNRs), an annual event, which brings nutrition stakeholders at National level to review the progress of implementation of nutrition actions in the country for the previous financial year.

## METHODS

### Design

This was a cross-sectional study, which employed qualitative method of data collection whereby semi structured interviews were used to collect data on the achievements, facilitating factors and challenges of multi-sectoral governance in improving child nutrition in Buhigwe District Council.

### Setting

This study was conducted in Buhigwe District, Tanzania, which is bordered to the north by Burundi, to the east by Kasulu Rural District and Kasulu Urban District, and to the west by Kigoma Rural District. According to the 2012 Tanzania National Census, the population of Buhigwe District was 254,342 and the population of under-five children was 62,208 in 2019. Buhigwe is one of the districts in Tanzania implementing the NMNAP for the period of 2015/16-2020/2021.

### Sampling and Sample Size

A sample size of 25 key informants were purposely drawn from a specific population of interest to the study-that is members forming the CMSCN and parents/caregivers who received significant multi-sectoral nutrition interventions for more than six months at community level. 17 CMSCN members were selected based on their occupational and professional experience and their involvement in multi-sectoral steering committee for nutrition, these were 1 - Council Medical Officer, 1 - Council Nutrition Officer, 1 - Council Planning Officer, 1 - Council Human Resource Officer, 1 - Council Treasurer, 2 Education Officers (Secondary and Primary), 1 - council Information Officer, 1 - Water Engineer, 1 - Council Community Development Officer, 1 - Council Livestock and Fisheries Officer, 1 - Council Agricultural, Irrigation and Cooperative Office, 1 - staff from nutrition focused Civil Society Organization, 2 - representatives from faith Based Organizations (one Christian & one Muslim), 1 - representative from media and 1 - representative from private sector. Eight (8) parents/caregivers were conveniently selected from the list of 76 parents/caregivers who were reached with messages to improve child nutrition and multi-sectoral interventions for more than six months. The selection of 8 parents/caregivers were based on their accessibility particularly geographical proximity. However, it is important to note that the final sample size for this study was determined after reaching saturation point.^18^

### Data Collection

The principal investigator (PI), conducted in depth interview with assistance from one research assistant who took notes and all non-verbal responses that emerged during the interview. A semi structured interview guide was used for conducting in-depth interviews with each study participant involved under the study. The focus of questions in the interview guide was divided into three thematic areas, namely achievements, facilitating factors and challenges of multi-sectoral governance in improving the nutrition status of children.

### Data Analysis

The qualitative data analysis employed a thematic approach which involved reading through the transcribed texts of each interview to identify responses relevant to the specific research questions of the study. Transcripts were read line by line to generate initial codes through data reduction. Recurrent themes were organized into subcategories then coherent categories to make meaning in the text. Themes were defined and named by refining the specifics of each theme and the overall story that the analysis tells to generate clear definitions and names for each theme basing on the study objectives.

### Ethical Considerations

Ethical approval for this study was obtained from Muhimbili University of Health and Allied Sciences Ethical Review Board with Ref.No. DA282/298/01.C/. The permission to conduct research was also approved by the District Executive Director for Buhigwe District Council. Before consenting, the moderator told the participants that their participation was purely voluntary. Furthermore, they were informed that no names were required and data would be treated with high level of confidentiality. All participants were informed about the purpose of the study and gave a written consent for their participation, audio recording and the anonymized publication of quotes for research purposes was assured. All measures to maintain the rights of human subjects in social research, including the right to privacy, confidentiality and prevent from any harm were considered.

### Trustworthiness

Several criteria were used in this study to evaluate the trustworthiness of the qualitative data. First, credibility was enhanced through spending a long period of time in the field to become familiar with the setting. Second, different perspectives of researchers in the study with different degrees of familiarity with the setting was included. Third, transferability is how applicable the findings are to other contexts.^19^ Here, the transcripts were translated from English to Kiswahili to increase the free expression of participants, and discussion of emerging themes among researchers enriched the interpretation of the data through the balance of perspectives representing different backgrounds and qualitative expertise. These measures enhanced the credibility of the representation of participants' views that is presented in our findings. Lastly, the detailed description of the study context, selection criteria, data collection and analytical process was complemented by quotations to allow readers to judge the dependability of the analysis and transferability of the findings.

## RESULTS

### Demographic characteristics

Table 1 provides a summary of the demographic information of participants who were involved in the study. A total of 25 key informants involved in the implementation of the multisectoral nutrition governance both at the council and community level participated in an in-depth interview. The actors at the council included implementers in the 6 public sectors including health, agriculture, education, water, livestock and fisheries and community development. Of the CMSCN who participated, 82% attended college and university education while 12% had A level education and 6% had primary education level. The majority of parents/caregivers who participated in the study had attained primary education (50%) while 13% never attended school.

**TABLE 1:** Summary of Socio-Demographic Characteristics of Study Participants

Characteristics	CMSCN members		Parents/Caregivers	
	Number	%	Number	%
	(n=17)		(n=8)	
Sex				
Male	13	76	1	13
Female	4	24	7	88
Age (years)				
25–34	3	18	1	13
35–44	6	35	2	25
> 45	8	47	5	63
Level of Education				
Never attended	0	0	1	13
Pre school	0	0	0	0
Primary	0	0	4	50
O-level	1	6	3	38
A-Level	2	12	0	0
College & University	14	82	0	0

Nine themes emerged from the stakeholder's perspectives regarding the multi-sectoral governance in improving child nutrition in Buhigwe District Council. These were further condensed to form three main categories basing on study objectives. These were achievements, facilitating factors and challenges of multi-sectoral governance in improving child nutrition in Buhigwe District Council.

### Achievements of Multi-Sectoral Governance in Improving Nutrition Status Among Children.

#### Allocation of specific budget for supporting nutrition interventions for under-five children.

Participants from health sector reported that they have allocated a budget for supporting nutrition intervention to under-five children in the council. The initiative is due to the establishment of the National Nutrition Compact between central government and regions which ensures allocation of nutrition funding per child (TZS 1000 per under-five child). Moreover, the continuous engagement of other sectors through the CMSCN and the annual multi-sectoral planning and budgeting meetings ensured proper allocation of nutrition funds across line sectors. However, the findings revealed that the disbursed budget from the council is inadequate to support planned activities as expressed by one of the participants:

*“We have allocated a budget of TZS 1000 per under-five child as one of the requirements in the performance contracts … however disbursed budget is still inadequate to support what we have planned” (participant #01, Government Official).*

Another participant added;

*“The CMSCN and annual multi-sectoral planning and budgeting meetings ensured proper allocation of nutrition funds across sector actors…” (participant # 11, Government Official).*

#### Improved Inter-sectoral coordination

Participants reported that all sector actors can sit and work together through a functional multi-sectoral steering committee for nutrition which tied all sectors together through CMSCN

“*Now we have a functional committee which is coordinated by the district executive director …. all sectors sit and work together to improve nutrition situation in our council.” (Participant # 15, Private Sector).*

#### Improved Knowledge

Parents/caregivers reported that their understanding on child feeding and practices had significantly changed after receiving the counselling on feeding practices through monthly support groups.

*“I used to feed my elder child with mainly ugali (hard porridge) and sweet potatoes…. after receiving counselling on feeding practices from monthly social groups, I have put more efforts in ensuring I feed my young child with varieties of food including fish, meat and fruits” (Participant # 21, Parent).*”

Another participant added:

*“I always thanks our local educator who told me to clean areas where my child plays and keep my chicken in a coop, my child used to play with chicken faces and I didn't know if they have any risk to my child…. now he doesn't have diarrhea cases and doesn't get sick often like before and I can see he has gained some weight” (Participant # 19, Parent).*

### Facilitating Factors for Improving Child Nutrition in the Context of Multi Sectoral Governance

#### Strong Leadership and Political Commitment

Participants reported the high commitment of the administrative authority of the council in tackling malnutrition in the council as one of the key facilitators in implementing nutrition interventions in the context of multisectoral governance. The council's authority was mentioned as among the potential actors in driving political commitment to address the malnutrition burden including ensuring allocation of budget for supporting interventions aiming at reducing malnutrition. Participants also reported that availability of enabling policies and guidelines which have facilitated implementation nutrition activities in the council as they help them to prioritize high impact activities to include in their action plans.

*“…Our district authority is very supportive for nutrition that's why many sectoral departments have allocated budgets to fight malnutrition…” (Participant # 09, Government Official)*.

Another participant added;

*“The availability of enabling policies and guidelines helps me to choose which priority activities to include in my annual plan, otherwise most of us would put activities with less impacts” (Participant #18, Private sector)*

Strong political commitment was also noted by the beneficiaries of the implementation of multisectoral nutrition interventions who reported that politicians have made nutrition issue part of their regular agenda.

*“We always see our district commissionaire coming to our cooking demonstration sessions and helping us to prepare nutritious food for our children” (Participant #26, Parent)*

#### Presence of NGOs Supporting Nutrition

The existence of NGOsworking in area of nutrition were reported to strengthen the multi-sectoral governance on nutrition since most of the interventions targeting under-five children have been receiving huge technical and funding support from NGOs.

*“…the government alone cannot get things done, NGOs provides funding and technical support on multi-sectoral governing areas like steering committees and supportive supervision.…” (Participant #12, Community Based Organization)*

#### Inter-Sectoral Linkages

Participants reported that the established structures to manage coordination across sectors is crucial to implement a multi-sectoral governance which will facilitate to reduce high burden of malnutrition.

*“…our regular participation in the multi-sectoral coordination meetings attended by government heads of departments is key in improving linkage among sectors so as to reduce malnutrition in our council…” (Participant #08, Faith Based Organization)*

### Challenges of Multi Sectoral Approaches in Improving Nutrition Status

#### Insufficient Financial Resources

Despite the fact that key informants reported that the multi-sectoral governance has assisted to have allocation of specific nutrition budget for supporting under-five children, it was also reported that the allocated budget is still inadequate. Insufficient financial resources were highlighted as a challenge for implementing a multi-sectoral governance programming in the study area.

*“We have allocated a budget of TZS 1000 per under-five child as among the key indicators in the nutrition compacts, however disbursed budget is still inadequate to implement all nutritional activities for under-five children” (participant # 16, Government Official).*

#### Inadequate Capacity

Respondents reported that they lack training, knowledge and skills in integrating nutrition within other sectors. Participants identified significant gaps in capacity for nutrition and emphasizes the urgency of the need for capacity-strengthening in the coordination structures especially CMSCN for effective multi-sector governance implementation.

*“As a member of the committee for nutrition, I am missing a formal knowledge on nutrition issues and it is not clear on what we are supposed to do and what to present during the meeting ” (Participant 15, Private Sector).*

Another participant added:

“*We are progressing well in implementing this plan but there is need to strengthen the capacity of the members of the CMSCN to understand nutrition and the role they need to play” (Participant # 05, Government Official)*

#### Poor Inter-Sectoral Financial Coordination

All sector actors are expected to allocate funds to implement interventions aiming at improving child nutrition in the council, however, such contribution from other sectors is not tracked and it's not clear how those funds are expended and managed. Thus, a cross-sectoral financing mechanism to track funding contribution specifically for child nutrition from other sectors was missing, in fact the different sectors are allocating their budget based on their own sectoral priority.

*“The council lack a cross sectoral financing mechanism to track the funding contribution from other sectors and it is not clear how those funds from other sectors are implemented and managed” (Participant # 05, Government Official).*

#### Lack of Necessary Tools to Collect Nutrition Information

There is an existence of Nutrition score card which is a tool to collect and manage nutrition data. However, the major nutrition indicators in the scorecard such as, wasting and stunting indicators are not collected due to lack of anthropometric tools at the health facilities to collect the required indicators.

*“When we fill scorecard you may find the empty spaces in the indicators for wasting and stunting, facilities are claiming to lack required tools to collect such information” (Participant # 01, Government Official)*

Another participant added:

*“I was told my child is stunted but the last time my child was measured height is when she was born, I cannot know my child growths status each time I visit health facility” (Participant #24, Parent)*

## DISCUSSION

This study has shown a number of achievements resulting from use of multi-sectoral governance in the implementation of nutrition activities in Tanzania. Such achievements include an increasing amount of funds allocated by the council to implement nutrition-related activities across different sectors, improved Inter-sectoral coordination and improved knowledge on child feeding and practices among community members. Furthermore, this study revealed that actors from all sectors can sit as one team and work together on nutritional activities through a functional multi-sectoral steering committee for nutrition which represents all sectors. These achievements were made possible because the senior officials at the council were held accountable by the compact agreement which was signed between them and the minister responsible for Regional Administration and Local Government Authorities on scaling up implementation of nutrition indicators especially the minimum mandatory nutrition funding allocation per child, (TZS 1000 per under-five child).^9^

Tanzania has made efforts to fuel action predominantly in the area of enabling environment within the NMNAP which aims to improve effectiveness and efficiency of nutrition governance (including coordination and leadership) and response across all sectors and actors.^8^ However less consideration has been given to explore the achievements, facilitating factors and challenges encountered by multi-sectoral governance for nutrition in Tanzania. A study of Nepal's nutrition governance capacity found improvement across all levels of administration in cross-sector engagement.^21^ However, it is also reported that the disbursed budget from the councils is still inadequate to implement all nutritional activities for under-five children. Despite the fact that the Minimum Nutrition Package is part of the recommended high impact intervention (HII), it has been noted that the budget allocated to it is underestimated and far below the proportion allocated to other interventions, with the Infant and Young Child Feeding Practices (IYCF) aspect frequently ignored.^21^

Parents/caregivers reported that that their ideas and perceptions of child feeding practices had significantly changed after receiving counselling sessions on feeding practices through monthly support groups' meetings aimed at improving child nutrition. The synergies between various multi-sectoral governance interventions and Tanzania Social Action Fund (TASAF) have contributed to over 40% TASAF beneficiaries been enrolled and regularly participate to monthly group counselling on optimal caregiving practices to improve maternal and child nutrition.^40^ An observational study on childhood stunting after exposure to multi-sectoral interventions (agriculture, income generation, improved water and sanitation, education, infrastructure) in 9 sub-Saharan African countries found a significant reduction in stunting prevalence in 5 of them.^26^ Despite an increased knowledge of parents/caregivers in the area of infant and young child feeding practices, the multi-sectoral team needs to combine a comprehensive package of services to parents/caregivers, including early stimulation, social and child protection, to support families providing nurturing care of children.

This study critically analysed potential facilitators for effective multi-sectoral nutrition governance towards improving child nutrition. Participants reported strong leadership and political commitment, presence of partner NGOs and inter-sectoral linkage as factors for improving child nutrition in the context of multi sectoral governance. In this study, the council's authority was mentioned as among the potential actors in driving political commitment to address the malnutrition burden including ensuring allocation of budget for supporting interventions aiming at reducing malnutrition. From this perspective, achieving political commitment is more than generating attention to malnutrition or getting it onto a government agenda. It further involves the mobilization of political systems and institutions, enabling policies, guideline, allocating resources and coordinating responses for as long as necessary to ensure positive results.^35^ Cascading multi-sectoral nutrition structures from central to local levels will depend on improved awareness, leadership and continued political commitment.^3^ Similarly, the participation of high-level politicians was seen as one of the key indications of political will and determination.^35,39^ The existence of inter-sectoral linkage was reported as important factor to implement a multi-sectoral governance which will facilitate the reduction of high levels of malnutrition especially to children. Establishing governance structures and policies to manage coordination across sectors is crucial to implementing a national Multi-Sectoral Nutrition (MSN) plan. The presence of development partners in nutrition such as NGOs were also reported as a facilitator which strengthens the multisectoral governance in improving nutrition outcomes. Effective engagement of development partners for nutrition in the MSN coordination structures have been found in Ethiopia to be useful in implementation of nutrition activities.^25^

Our findings confirm that key challenges to effective multi-sectoral governance is insufficient financial resources. Studies conducted in Ethiopia and Burkina Faso have also documented the insufficient financial resources as the main challenges of the implementation of activities involving multisectoral collaboration. ^16,25,37^ Furthermore, participants reported lack of a cross-sectoral co-financing mechanism. This could be explained with the fact that different sectors have different priorities and are allocating their budget based on their own sectoral priority. Thus, tracking funding contributions and performance of sector actors in implementing nutrition sensitive interventions in the councils is a challenge. Consequently, the sectors, in carrying out programs to fulfill their particular mandates, inevitably situate themselves as competitors for resources rather than as partners in action.^3^ Multi-sectoral approaches can suffer due to differing cultures, mandates, and incentives characteristic of different sectors. These factors are barriers to coordination and cooperation, and turf battles further interfere with integrated action.^28^ The major nutrition indicators such as wasting and stunting indicators are not collected due to lack of anthropometric tools to collect the required indicators. Moreover, it is reported that CMSCN's members lack training, knowledge and skills in integrating nutrition within sectoral departments. Participants identified significant gaps in capacity for nutrition and emphasizes the urgent need for capacity-strengthening in the coordination structures for effective multi-sector governance implementation. A district director of health services in Uganda noted that local political leaders first ask for the construction of health units in seeking to find a solution to the health problems their constituents face. However, she noted that, they rarely go a step further to consider what are the causes of those health problems, including malnutrition. A knowledge gap must be bridged if nutrition is to become a more significant part of the content of local government debate, planning, and action.^3^ Lack of knowledge or commitment to nutrition, lack of resources and presence of competing priorities within individual sectors were identified as barriers to effective coordination between health and agriculture sectors.^37^ There is a general need for nutrition awareness in the area, as relates to basic nutrition-sensitive roles and responsibilities of the members in the CMSCN. This is similar to a recent analysis conducted of the African Nutrition Security Partnership, which identified a broad range of governance challenges encountered by African countries, including human resource constraints for overall MSN implementation and coordination, lack of dedicated implementation staff for subnational efforts, and failure to engage high-level decision-makers.^32^

### Study Limitation and Mitigation:

The main limitation of this study is the selection of only one district for the study, which may have limited the information generated about the implementation of multi-sectoral governance in improving child nutrition. However, the researchers used triangulation methods to collect data and they have ensured that data collected from this district reached saturation point, thus provided adequate and detailed information to answer the research questions.

## CONCLUSION AND RECOMMENDATIONS

Smooth implementation of nutrition interventions targeting undernourished under-five children require strong multi-sectoral governance, which is supported by adequate spending of the allocated funds, political commitment, operationalized policies and guidelines for nutrition. However, key stakeholders including central and local government authorities should address the challenge of inadequate capacity, lack of a cross-sectoral co-financing mechanism and lack of necessary tools to collect nutrition information, which were reported as barriers to the implementation of multi-sectoral governance in improving child nutrition. Strengthening sector participation and mobilizing financial resources have the potential to significantly reduce barriers and improve the quality of implementation of multi-sectoral governance in improving child nutrition. Further research that focuses on multi-sectoral governance at the ward and village level, as the lowest administrative levels of the Tanzania Local Government System, is also recommended.
